# POSITIVE ACTIVITIES AND WELL-BEING AMONG DEMENTIA CAREGIVERS: MODERATING EFFECTS OF CAREGIVER BURDEN

**DOI:** 10.1093/geroni/igad104.1914

**Published:** 2023-12-21

**Authors:** Kira Birditt, Angela Turkelson, Akari Oya, Crystal Ng, Courtney Polenick

**Affiliations:** Institute for Social Research, University of Michigan, Ann Arbor, Michigan, United States; University of Michigan: Institute for Social Research, Ann Arbor, Michigan, United States; University of Michigan, Ann Arbor, Michigan, United States; University of Michigan, Ann Arbor, Michigan, United States; University of Michigan, Ann Arbor, Michigan, United States

## Abstract

ADRD caregiving is common and often predicts increased caregiver burden which has negative implications for mental and physical health. Thus, it is important to understand the modifiable factors that may lead to less burden among caregivers. Positive activity theory and research suggest that engaging in positive activities may lead to greater positive and less negative affect and less burden. The purpose of this study was to examine positive activities and positive social interactions among ADRD caregivers and their links with well-being. A total of 98 caregivers completed baselines and EMAS every three hours for 5 days. Greater positive activities and more positive interactions with PLWD predicted greater positive affect, less negative affect, and lower daily caregiver burden. Mediation models indicated that positive and negative affect fully accounted for the links between positive activities and caregiver burden and partially accounted for the links between positive interactions with PLWD and caregiver burden. These effects were greater among caregivers who reported greater overall burden at baseline. There were no such effects when examining positive interactions with social ties other than the PLWD. Thus positive activities and positive social interactions with PLWD may represent an important target for future interventions with an identified mechanism of action (affect) and demonstrated benefit for daily burden among high risk caregivers.

